# One in a Million: A Case Report of Stiff Person Syndrome

**DOI:** 10.1155/2022/7741545

**Published:** 2022-01-13

**Authors:** Ruchi Yadav, Neeraj Abrol, Sima Terebelo

**Affiliations:** ^1^Department of Medicine, One Brooklyn Health, Brookdale University Hospital, Brooklyn, NY, USA; ^2^Division of Rheumatology, One Brooklyn Health, Brookdale University Hospital, Brooklyn, NY, USA

## Abstract

Stiff person syndrome (SPS) is a rare autoimmune disease caused by lack of inhibition to excitatory neurotransmitters in the central nervous system (CNS) leading to inappropriate motor unit firing. The pathophysiology is incompletely understood; however, high titers of antiglutamic acid decarboxylase antibody (anti-GAD Ab) are strongly associated with this disease. We present a 50-year-old woman with a history of ongoing gait and balance issues for 5 years with multiple negative workups. She recently had an acute exacerbation which left her bedbound, unable to move her legs or turn from side to side. After a negative workup at an outside hospital, the patient was discharged to a subacute rehabilitation facility. She then presented to our institution due to worsening of her condition and was ultimately diagnosed with SPS which was successfully treated. We review the case presentation and treatment options in the context of a severe disabling disease presentation.

## 1. Introduction

SPS is a rare autoimmune disease caused by lack of inhibition to excitatory neurotransmitters in the CNS, leading to continuous involuntary muscle excitation [1]. It has various presentations, including incomplete localized stiffness in only one limb (stiff limb syndrome, SLS) to fulminant stiffness with encephalomyelitis, brainstem dysfunction, and dysautonomia (progressive encephalomyelitis with rigidity and myoclonus, PERM) [1]. SPS course varies; some patients have slowly progressive disease, others have acute exacerbations, and others may switch from one phenotype to another [2]. This adds to the diagnostic challenge. Many patients are initially suspected of psychogenic stiffness or malingering. It is important to consider SPS in a patient with stiffness and apparent resistance to passive range of motion on exam as SPS is an autoimmune-mediated illness which is treatable if diagnosed [2]. We report a case of a patient who had a history of difficulty walking with reported gait and balance issues for 5 years with multiple negative work ups by her primary care physician and outside consultants. She was diagnosed after an acute exacerbation left her incapacitated, unable to move from her bed. One month after initiating immunosuppressant treatment and rehabilitation, the patient was back home, walking with a rolling walker, at about 75% of her prior functional capability and was continuing to improve with ongoing home-based physical therapy, symptomatic treatment, and immunosuppression.

## 2. Case Presentation

The patient is a 50-year-old woman, formerly from Jamaica, with no significant medical history who presented to our hospital with chronic lower back pain (LBP), acute stiffness of her bilateral lower limbs, and inability to walk. Her symptoms first began 5 years ago when she started having chronic LBP and bilateral knee pains which progressed to involve right leg stiffness and frequent falls. She had a negative workup by her primary care physician and specialists at an outside hospital; however, her symptoms continued to progress despite physical therapy and ibuprofen. One month prior to admission, the patient noticed an acute onset of stiffness in her bilateral lower limbs. She presented to an outside hospital with complaints of inability to ambulate, tingling and numbness in her bilateral lower extremities, lower back pain, 30 lb weight loss, chills, and sweating. At that time, physical exam was significant for tenderness to palpation of the bilateral lower legs from the knee to the ankle and inability to assess range of motion or muscle strength in the bilateral knees and hips due to pain and weakness. It was noted that plantar flexion strength was preserved 5/5. Physical therapy evaluation noted that moderate assistance was needed for the patient to roll to either side; while supine, maximal assistance was required to move from supine to sit, and the patient was unable to stand and ambulate secondary to bilateral lower extremity spasms and LBP. An extensive workup was done and was significant for antinuclear antibody (ANA) 1 : 160, erythrocyte sedimentation rate (ESR) 44 mm/h (0–40), C-reactive protein (CRP) 27.7 mg/L (0–4), creatinine kinase (CPK) 1553 U/L (20–180), and thyroid stimulating hormone (TSH) 4.620 uIU/mL (0.270–4.200). Atypical antineutrophil cytoplasmic antibody (ANCA) was indeterminate; however, proteinase 3 ANCA and myeloperoxidase ANCA were both negative. The remaining laboratory results were negative or within normal limits, including white blood cell (WBC) count, interferon gamma release assay (IGRA), human immunodeficiency virus (HIV), Lyme antibodies, human T lymphotropic virus type I/II (HTLV I/II) antibodies, neuromyelitis optica (NMO) antibodies, serum protein electrophoresis, Smith, ribonucleoprotein (RNP), complement C3 and C4, Sjogren antibodies (SSA, SSB), rheumatoid factor, anticitrullinated peptide antibodies, serum copper, and serum vitamin E.

Noncontrast magnetic resonance imaging (MRI) of the thoracic and lumbar spine showed evidence of abnormal signal within the right side of lower thoracic cord extending T10–T12, as well as some mild to moderate degenerative changes. Other imaging studies included a thyroid ultrasound which showed multiple thyroid nodules and a computed tomography (CT) scan of the chest, abdomen, and pelvis with contrast which showed focal subcentimeter hypoattenuating cystic structures (hepatic cysts versus focal biliary ductal dilatation) and mild pelvic ascites. The patient was discharged to a subacute rehabilitation facility with planned neurology follow-up as an outpatient.

Three weeks later, the patient presented to our hospital due to worsening of her generalized stiffness. At that time, the patient was bedbound, unable to sit or flex her legs. She reported ongoing LBP, worsening stiffness in her lower limbs most pronounced at the knee joints, and continued weight loss with occasional episodes of subjective fever and chills. Review of systems was otherwise negative. The patient denied smoking or recreational drug use and had no family history of autoimmune or neurological disease. She had never had anything like this before.

On exam, the patient was afebrile with normal vital signs, alert, and oriented to self, time, and place with no aphasia or dysarthria. Heart, lung, and abdomen exams were benign. Musculoskeletal exam did not show any synovitis, effusions, or tenderness to palpation of any joints. Range of motion was normal in the upper extremities but was unable to be assessed in the hips or knees due to stiffness and inability to flex actively. Attempted passive knee flexion caused quadriceps contraction and was painful for the patient. The hips could be passively flexed to 30 degrees bilaterally, limited by pain and stiffness. The patient was unable to turn to either side to assess her spine and could not sit up. The big toe was noted to be dorsiflexed bilaterally and could not be passively plantarflexed. Neurologic exam was significant for normal cognitive function, normal cranial nerve assessment, normal sensory exam, and normal cerebellar function. Motor strength was 5/5 in bilateral upper limbs (proximally and distally). Proximal lower limb muscle strength could not be adequately assessed. Distal lower leg strength was 5/5 on foot dorsiflexion and plantar flexion. Patient was noted to be able to hyperextend her knees and thus lift her calves up slightly. She could not flex her hips or knees. Deep tendon reflexes (DTR) were 1+ in both biceps, triceps, and brachioradialis. DTR were absent in both the knees and ankles. Plantar responses could not be assessed as the big toe was persistently dorsiflexed bilaterally.

Initial laboratory evaluation was significant for ESR 80 mm/h (0–30), CRP 2.2 mg/dL (0.5–1), negative ANA, no leukocytosis, and normal kidney function. Lumbar puncture showed clear, colorless cerebrospinal fluid (CSF) with negative xanthochromia, WBC 24, lymphocyte 20, monocytes 4, and RBC 14, with normal protein and glucose. Further serologic testing was done, with negative or within normal limit results for WBC, ANA, IGRA, procalcitonin, angiotensin converting enzyme (ACE), CPK, HTLV I/II, and TSH. Anti-GAD Ab was >25,000 U/mL (0–5), supporting the suspected diagnosis of SPS.

**Figure 1:**
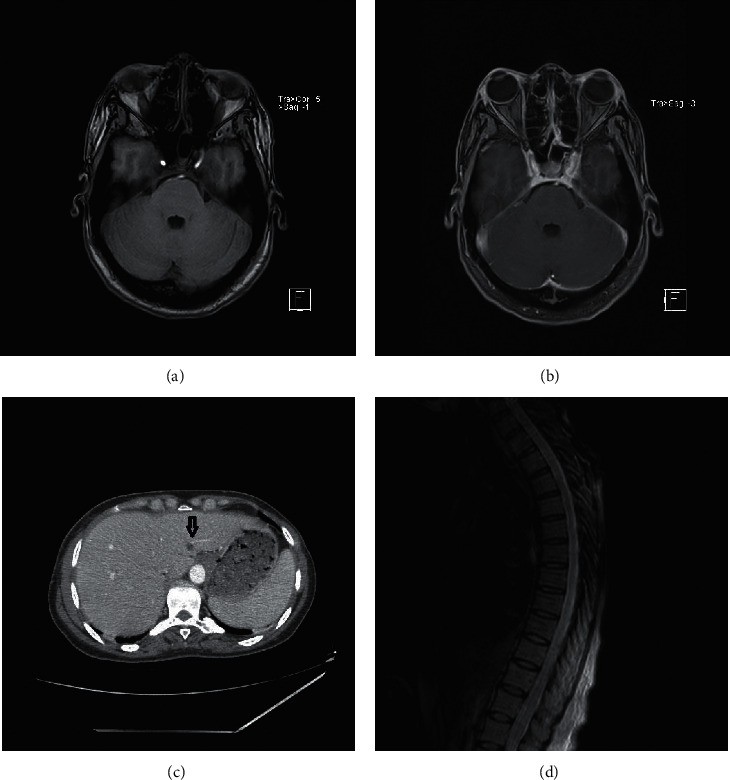
(a) MRI brain precontrast. (b) MRI brain postcontrast, showing dural enhancement. (c) CT chest, abdomen, and pelvis showing the hypoattenuating nodule in the liver (arrow). (d) MRI lower thoracic spine showing mild degenerative changes.

Repeat MRI imaging of the cervical, thoracic, and lumbar spine with contrast showed multilevel degenerative changes with no abnormal signal, no evidence of inflammation, mass or abscess in the spine, or paraspinal muscles. MRI brain with contrast showed nonspecific mild dural enhancement. Repeat thyroid ultrasound again showed multinodular thyroid, which was assessed by endocrinology as benign and not requiring any current intervention (Figure 1).

There remained concern for paraneoplastic syndrome, and further workup was sent, as well as labs to rule out associated autoimmune conditions. Glycine receptor antibodies were high positive. Thyroid peroxidase antibodies were 350 IU/mL (0–34). Remaining labs were negative or within normal limits, including dipeptidyl peptidase antibodies, amphiphysin antibodies, antiparietal cell antibodies, vitamin B12 levels, methylmalonic acid and hemoglobin A1c. The patient was also assessed with another CT scan of her chest, abdomen, and pelvis which did not show any signs of malignancy.

The patient was initially treated with gabapentin and baclofen; however, there was no significant improvement in her stiffness. Baclofen was discontinued and diazepam was started along with inpatient physical therapy with some initial improvement in spasticity. The patient was treated for SPS with intravenous immunoglobin (IVIG) 2 g/kg given over 5 days with continued improvement. She then was given solumedrol 1 g daily × 3 days with more improvement and finally was given rituximab 1000 mg with continued improvement in her spasticity. Prior to discharge, the patient could turn in her bed unassisted, could flex her left hip and knee fully, could flex right hip fully and right knee with assistance to 110 degrees, and could stand with assistance. Her Rt big toe remained dorsiflexed, but she could now wiggle it independently. The patient was discharged to acute rehabilitation on gabapentin 200 mg three times a day, diazepam 5 mg every 6 hours, and prednisone 60 mg/d with plans for monthly IVIG infusions, a second rituximab dose at 14 days after the initial dose and outpatient prednisone taper.

The patient is currently 3 months posthospitalization and has progressed from being bed bound and unable to turn in her bed to now being able to stand and walk with a rolling walker. Her right knee remains somewhat stiffer than her left. Steroid dose is being tapered, and she is planned to continue IVIG monthly, rituximab every 6 months, diazepam, and gabapentin.

## 3. Discussion

SPS is a rare disease with an estimated prevalence of 1-2 cases per million [1]. The pathophysiology is incompletely understood; however, high titers of anti-GAD Ab are strongly associated with this disease and immunosuppressive treatment helps improve the muscle stiffness [2, 3]. There is underlying central inhibition of inhibitory signals which ultimately manifests as inability of opposing skeletal muscles to relax when the contralateral muscles contract, leading to the characteristic rigidity and painful spasm [3]. SPS has various presentations including classic SPS, partial SPS, and paraneoplastic SPS, all of which share the characteristic symptom of muscle stiffness. Classic SPS, first described in 1956 [4], is described as diffuse stiffness, truncal rigidity, and muscle spasms in the setting of anxiety and exaggerated startle with symptoms precipitated by sudden stimuli. Partial SPS only affects part of the body, most commonly “stiff limb syndrome,” which affects the legs. It may present with more spasticity in one leg than the other and causes difficulty walking. Paraneoplastic SPS is seen in approximately 5% of cases. It looks identical to classic SPS but usually is anti-GAD Ab negative and has positive amphiphysin antibodies. It tends to be resistant to immunomodulatory treatment [2].

SPS is notoriously difficult to diagnose as it is rare, uncommonly considered, and symptoms evolve over time. A retrospective cohort of 23 patients with SPS spectrum disorders reported that patients were diagnosed on average 3.5 years after initial symptoms and first began immunomodulatory treatment with IVIG more than 5 years after symptom onset [5]. The initial presenting symptoms, as assessed by Tsiortou et al. and colleagues in a retrospective cohort of 57 patients, were slowly progressive proximal leg stiffness which then progressed to muscle spasms, stiffness in the thoracolumbar spine, muscle rigidity, and hyperreflexia [3]. Many patients were noted to have comorbid anxiety due to fear of falling and the unpredictable nature of the muscle spasms, leading to erroneous diagnosis of a psychogenic disorder [3].

Our patient too presented with a history of slowly progressive right lower extremity weakness and gait disturbance over 5 years, with multiple negative work ups. She then had a recent acute exacerbation with subsequent hospitalizations and concomitant workup. The acute decompensation raised diagnostic considerations including a possible psychogenic component to the patient's stiffness and inability to move. Other diseases under consideration were HTLV I/II myelopathy based on her presenting symptoms of painful spastic paraparesis without sensory loss in a middle age woman of Caribbean origin, HIV myelopathy, secondary Parkinsonism of lower limb vascular Parkinson's, and neurosarcoid. This struggle to identify the diagnosis is concordant with the described SPS cohorts, in which most patients were first misdiagnosed. The most common erroneous diagnosis was conversion disorder or other psychiatric disorders. Other common initial diagnoses were Parkinsonism, myelopathies, and dystonia [3].

The diagnosis of SPS is challenging and relies on a combination of clinical findings, antibody testing, electrophysiologic testing, CSF analysis, and imaging to rule out structural lesions and other possible causes. There also is a role for a malignancy work up to rule out the paraneopastic cause of SPS [2, 3]. Most patients will have stiffness of axial muscles, high anti-GAD ab, symptoms of anxiety, and electromyographic (EMG) findings of continuous motor unit firing in the absence of any other physiologic cause after extensive investigation. Anti-GAD Ab is pathognomonic; another commonly associated autoantibody is glycine-a1 receptor Ab (anti-GlyR), which is also found in progressive encephalomyelitis with rigidity and myoclonus (PERM) [3]. Our patient had the characteristic truncal and LE rigidity and high anti-GAD ab; however, she did not have anxiety. EMG was not available at the time of her hospitalization; however, given the otherwise negative diagnostic workup, high titer anti-GAD ab, and the positive anti-GlyR, we were able to diagnose and treat her.

Therapy for SPS is a combination of symptomatic treatment and immunosuppression. Gamma aminobutyric acid (GABA) agonists such as benzodiazepines, gabapentin, baclofen, and pregabalin provide immediate relief and help mobilize the patient quickly. Immunomodulators address the underlying autoimmune defect and provide lasting benefits; however, therapy is dictated by symptom severity. IVIG has been used with much success and is considered the first-line treatment for SPS [2, 6]. A randomized crossover trial assessing IVIG therapy versus diazepam found that IVIG was more effective in relieving muscle stiffness. When IVIG was stopped, the patients experienced a rebound worsening of their stiffness [7]. Reports of long-term follow-up of patients on IVIG therapy show that the patients remained stable and continued to improve clinically even after 3–5 years of therapy [5].

Patients with more acute presentations or marked impairment may benefit from more aggressive immunosuppression. High-dose steroids, plasmapheresis, various steroid sparing immunosuppressants, and rituximab all have been tried with varying degrees of efficacy. Our patient had no response to baclofen but a slight response to diazepam combined with gabapentin. She had an encouraging response to IVIG; however, still remained markedly impaired. Given the acuity of her symptoms, high inflammatory markers, and signs of inflammation in the CNS, the patient was given pulse steroids with further improvement. She then received rituximab with hopes for even more improvement towards our goal of returning her to her former functional status of ambulatory with a cane.

The data on rituximab are mixed. Several case reports describe remarkable improvement in patients who were given rituximab infusion to treat SPS. A review of case reports describing patients with refractory SPS already on standard of care found that there was an increase in SPS remission with increasing rituximab dose and frequency [8]. A recent randomized controlled trial in a cohort of 24 patients overall did not show statistically significant improvement with rituximab use. However, there was sustained improvement in a subset of 4/12 pts who were given the active drug. Additionally, the protocol provided for one cycle (2 doses of 1 g each) of rituximab with follow-up observation over 6 months [9]. A more recent abstract of long-term use of rituximab in a 41-patient observational cohort showed long-term benefit in 74% of the patients who had long-term follow-up [10]. In this cohort, 17/23 patients who were on chronic rituximab infusions showed improvement in SPS symptoms, ranging from resolution of axial rigidity to objective improvement in gait [10].

## 4. Conclusion

In conclusion, SPS is a rare disease with multiple presentations. It remains an important consideration in the differential diagnosis of muscle stiffness as correct treatment can arrest the course of the disease and reverse some of the disabilities. It is vital to consider unusual presentations of rare diseases and test to exclude all possible organic causes prior to diagnosing a patient with psychogenic disease.

## Data Availability

No data were used to support this study.
